# Prostate Cancer Mortality in Men not Attending a Population-based Screening Program: The ‘Good’, the ‘Bad’, and the ‘Ugly’ After 20-yr Follow-up in the European Randomised study of Screening for Prostate Cancer

**DOI:** 10.1016/j.euros.2026.06.009

**Published:** 2026-07-30

**Authors:** Renée C.A. Leenen, Esmée F.H. Mulder, Sebastiaan Remmers, Ivo I. de Vos, Kirsi Talala, Jonas Hugosson, Rebecka Arnsrud-Godtman, Elly Den Hond, Arnauld Villers, Gregoire Pionas, Luis Llane, Marcos Lujan, Giuseppe Gorini, Donella Puliti, Maciej Kwiatkowski, Stephen Wyler, Chris Bangma, Fritz H. Schroder, Anssi Auvinen, Monique J. Roobol, ERSPC Study Group

**Affiliations:** a Erasmus MC Cancer Institute, University Medical Center Rotterdam, Department of Urology, Rotterdam, The Netherlands; b Cancer Society of Finland, Helsinki, Finland; c Department of Urology, Institute of Clinical Sciences, Sahlgrenska Academy at the University of Göteborg, Göteborg, Sweden; d Provincial Institute of Hygiene (PIH), Antwerp, Belgium; e Department of Urology, CHU Lille, University Lille Nord de France, Lille, France; f Department of Urology, Clinique Beau Soleil, Montpellier, France; g Department of Urology, Hospital Universitario de Getafe, Madrid, Spain; h Department of Urology. Hospital Universitario Infanta Cristina, Madrid, Spain; i Oncologic Network, Prevention and Research Institute (ISPRO), Florence, Italy; j Research Coordination Unit, Meyer Children’s Hospital IRCCS, Florence, Italy; k Department of Urology, Cantonal Hospital Aarau, Aarau, Switzerland; l Faculty of Medicine, University of Basel, Basel, Switzerland; m Department of Urology, Academic Hospital Braunschweig, Braunschweig, Germany; n Prostate Cancer Research Center, Faculty of Social Sciences, University of Tampere, Tampere, Finland

**Keywords:** Early detection of cancer, Follow-up studies, Prostate cancer, Prostate-specific antigen

## Abstract

**Background and Objective:**

Nonattendance can substantially decrease the effectiveness of existing and potential future population-based screening programmes for (prostate) cancer*.* We evaluated the long-term association between nonattendance and prostate cancer–specific mortality (PCSM) in the European Randomised Study of Screening for Prostate Cancer (ERSPC), with up to 20 yr of follow-up.

**Methods:**

This secondary analysis included 161 380 men aged 55–69 yr randomised to receive invitations for Prostate-specific antigen-based (PSA)-based screening (*n* = 72 460, screening arm [SA]) or to the control arm (CA) (*n* = 88,920) across seven ERSPC centres. Within the SA, men were classified as screening never-attenders (no attendance at any screening round) or screening ever-attenders (attended at least one screening round). The primary endpoint was PCSM. Cumulative incidence of PCSM was calculated using competing risk analysis, accounting for death from other causes as a competing event. Poisson regression was used to estimate rate ratios (RR) of cumulative PCSM between three groups: screening never-attenders, ever-attenders, and CA.

**Key Findings and limitations:**

Of the 72 460 men in the SA, 12,401 (17%) were defined as never-attenders. At 20 yr, the cumulative PCSM was 1.4% (95% confidence interval [CI], 1.2–1.6) among never-attenders, 1.2% (95% CI, 1.2–1.3) in the CA, and 1.0% (95% CI, 0.9–1.0) in ever-attenders. For PCSM, we observed an RR of 1.39 (95% CI, 1.2–1.6; *p* < 0.001) for never-attenders compared to the CA, while for ever-attenders compared to the CA, we observed an RR of 0.77 (95% CI, 0.69–0.85; *p* < 0.001). Data on determinants of attendance are lacking.

**Conclusions and Clinical Implications:**

Men randomised to the SA but who never attended had higher observed PCSM than men in the CA who were not offered screening. Conversely, men in the SA who attended screening had lower observed PCSM than men in the CA, with a larger observed difference than that reported in prior intention-to-screen analyses.

***Trial registration:*** ISRCTN49127736.

## Take Home Message

While both screening never-attenders and men in the control arm did not undergo organised Prostate-specific antigen-based screening, we observed a higher risk of dying from prostate cancer for the former than the latter.

ADVANCING PRACTICE **What does this study add?**

This is the first ERSPC analysis to observe the long-term impact of screening never-attendance on prostate cancer–specific mortality across all seven centres. In the context of low, variable, and declining participation in contemporary prostate cancer screening trials and pilots, as well as established population-based screening programmes for other cancers, this study provides insight into the consequences of nonattendance and highlights its role in creating health inequities.

**Clinical Relevance**

Nonattenders derive no benefit from organised PSA screening because they are never exposed to the early detection pathway that screening provides. Moreover, nonattendance likely identifies a distinct subgroup of men with lower healthcare engagement and different health-seeking behaviours, placing them at even greater risk of prostate cancer mortality than the average unscreened population, in whom opportunistic PSA testing is common. These findings highlight that improving participation in organised screening programmes may be as important as optimising the screening strategy itself to maximise reductions in prostate cancer mortality. Associate Editor: Roderick C.N. van den Bergh.

**Patient Summary**

We analysed long-term outcomes in a large European prostate cancer (PCa) screening trial. For men who were invited for PCa screening using a prostate-specific antigen test but never attended, we observed a higher risk of dying from PCa than we observed for men who were not invited or who attended screening.

## 1 Introduction

Prostate cancer (PCa) remains a major health burden worldwide, with the number of new PCa cases expected to double in the next 20 yr [1]. The European Randomised study of Screening for Prostate Cancer (ERSPC) was initiated in the early 1990s to assess the effect of prostate-specific antigen (PSA)-based screening on PCa-specific mortality (PCSM) at a population level [2], [3]. The ERSPC has consistently demonstrated a relative risk reduction of PCSM associated with PSA screening [3]. Now, more than 30 yr after the initiation of the ERSPC, countries are piloting the implementation of risk-stratified, population-based screening programs for PCa early detection [4], [5], [6].

However, differences between trial conditions and real-world practice create inevitable efficacy–effectiveness gaps. A key potential contributor is nonattendance, which can significantly influence the benefits of organised population-level screening. For any screening programme to be effective, the World Health Organization (WHO) recommends that 70% of the population needs to be covered by screening [7]. Nevertheless, contemporary PCa screening trials, as well as established population-based screening programmes including those for other cancers, face low, varying, and even declining participation. In ongoing PCa screening trials, participation ranges from 11% to 52% [8], [9], [10], [11], [12]. In established cancer screening programs such as colorectal cancer screening, the participation proportion is above 70% in only one out of 18 European Union member states (EU-MS) and less than 40% in 10 EU-MS. For breast cancer screening, participation varies across EU-MS, ranging from over 80% to less than 30%, with 13 MS reporting declines between 2012 and 2022 [13]. A recently published analysis of the Swedish Mammography Screening programme demonstrated that women who did not attend their first screening invitation—representing 32% of all women invited—demonstrated a 40% higher long-term breast cancer mortality risk compared with attenders [14]. These participation issues impose a major challenge for the success of existing and potential future nationally-organised screening programmes for (prostate) cancer. This underscores the need to better understand the impact of nonattendance in population-based screening programmes.

As such, we aimed to evaluate the association between screening attendance and long-term PCSM. To do so, we estimated the risk of PCSM in men who were randomised to the screening arm (SA) but did not attend any of the screening rounds, so-called screening never-attenders, as compared to men in the control arm (CA) using long-term data from the ERSPC. Additionally, we estimated the risk of PCSM in SA ever-attenders, as compared to men in the CA, to descriptively estimate PCSM in men who did attend screening, without inferring effectiveness and causality.

## 2 Materials and methods

### 2.1 Trial design and study population

The ERSPC study protocol and population have been previously described [2], [15], [16]. The current analysis includes 72 460 and 88 920 men aged 55**–**69 yr at entry (core age group) randomised to a SA and CA, respectively, across seven ERSPC centres. The centres include Finland, the Netherlands, Italy, Sweden, Switzerland, Belgium, and Spain. Randomisation started in 1993 with the Netherlands and Belgium, followed by the other centres between 1993 and 2003.

Participants were assigned to study arms using a 1:1 allocation ratio, except for the Finnish centre, which randomised annually 8000 men to screening, and the rest of the target population formed the CA, resulting in approximately a 1:1.5 ratio (SA: CA). The type of randomisation (before or after consent) differed across centres. Sweden, Finland, and Italy employed a Zelen-type effectiveness design, randomising men before obtaining consent, whereas the remaining centres used an efficacy design, randomising men after consent was obtained. Across these seven centres, participants were offered between one and ten screening rounds, with screening intervals ranging from 2 (Sweden) to 7 (Belgium) yr, depending on national protocols [15], [16].

### 2.2 Definitions

A man randomised to the SA who did not attend any of the offered screening rounds was defined as a screening never-attender. These men were included in the SA of the main ERSPC trial reports as part of the intention-to-screen analysis. Men randomised to the SA who attended at least one screening round were classified as screening ever-attenders. Men in the CA were not offered screening, and thus, attendance status does not apply to them.

### 2.3 Outcome

The primary outcome is PCSM. Within the ERSPC, the cause of death is determined by a Cause of Death Committee, which evaluated all medical records using a fixed algorithm or by the use of death certificates from official statistics, provided that a good agreement was shown with the Committee assignment [17], [18].

### 2.4 Statistical analyses

For the current analysis, follow-up was truncated on 31 December 2020, with a maximum follow-up of 20 yr. Men who had been diagnosed with PCa before randomisation were excluded. We adjusted for immortal time bias by subtracting the number of days between randomisation and first screening attendance from the total time between randomisation and death or PCa diagnosis. For ever-attenders of screening, this was calculated at an individual level. For screening never-attenders – who by definition did not attend any screening round - this information was unavailable. Therefore, their immortal time was estimated using the mean number of days between randomisation and the first screen of the attenders per centre. Consequently, the index time differed across groups: for screening ever-attenders, the index time was the first screening visit; for screening never-attenders, the index time was the assumed ‘first’ screening visit; and for the CA, the index time was years since randomisation. To assess the robustness of the immortal time bias correction, a sensitivity analysis was conducted in which a symmetric correction was applied to all groups.

Using competing risk analysis, thereby accounting for death from other causes as a competing event, the cumulative incidence of PCSM was estimated for the three defined groups: (1) screening never-attenders, (2) screening ever-attenders, and (3) the CA. Poisson regression was used to estimate rate ratios (RRs) for PCSM between the three groups, using the logarithmic transformation of the follow-up as an offset.

### 2.5 Sensitivity analyses

We expected that the method of randomisation (before or after consent) had a strong effect on the proportion of never-attenders, with the highest proportion expected in centres using randomisation before obtaining consent. Therefore, to evaluate the effect of type randomisation for the three groups on PCSM, we conducted sensitivity analyses by updating our abovementioned Poisson regression model. First, we defined a model including main effects for both the three defined groups and the type of randomisation. Second, we used a model including an interaction term between the three groups and the type of randomisation. Differences in goodness of fit between these models and the ‘original’ Poisson regression model were assessed with a likelihood ratio test. Additionally, we conducted subgroup analyses for the type of consent and attendance. To assess the potential influence of centre-specific effects, centre was added as a covariate to the primary Poisson regression model in a separate sensitivity analysis. Lastly, to assess the robustness of the immortal time bias correction, a sensitivity analysis was conducted in which a symmetric correction was applied to all groups.

All statistical analyses were conducted using R Statistical Software version 4.4.0.

## 3 Results

### 3.1 Study population and attendance

Of the 72 460 men randomised to the SA, 12 401 men (17%) did not attend any of the offered screening rounds and were classified as screening never-attenders, whereas 60 059 men (83%) attended at least one screening round (screening ever-attenders) (see Table 1). The proportion of never-attenders in the SA varied across centres, influenced by the method of randomisation. Centres employing pre-consent randomisation (Zelen’s design)—namely Finland (25%), Sweden (24%), and Italy (21%)—had higher proportions of never-attenders. In contrast, postconsent centres had lower proportions of never-attenders in the screening arm: the Netherlands (5.3%), Belgium (9.2%), Switzerland (2.2%), and Spain (0%). As a result, 88% of all screening-never-attenders originated from the three pre-consent centres (see Table 2). Among screening ever-attenders, the median number of screening visits was 2 (interquartile range [IQR], 1.5–3.0). Table 1 shows the baseline characteristics and the cumulative incidence of PCa and PCSM at 20 yr (including RRs) for the overall study population. The median follow-up among men without a PCSM event was 20 yr (IQR, 15.6–20.0) for ever-attenders, 20 yr (IQR, 14.3–20.0) for the CA, and 16.8 yr (IQR, 7.6–20.0) for never-attenders.

**Table 1:** Baseline characteristics, PCa detection and mortality within 20 yr, and rate ratios by group

	Never-attenders	Ever-attenders	Control arm
*n* (%)	12 401 (7.7)	60 059 (37)	88 920 (55)
Age at randomisation (yr), median (IQR)	59 (56–63)	60 (57–64)	59 (56–64)
PCa detection at 20-yr, cumulative incidence (95% CI) [*n*]	8.4 (7.9–8.8) [1035]	15 (14–15) [8712]	11 (11–11) [9529]
PCa specific mortality at 20-yr, cumulative incidence (95% CI) [*n*]	1.4 (1.2–1.6) [175]	1.0 (0.9–1.1) [578]	1.2 (1.2–1.3) [1105]
RR vs control (95% CI)	1.39 (1.2–1.6)	0.77 (0.69–0.85)	Reference

CI = confidence interval; IQR = interquartile range; PCa = prostate cancer.

**Table 2:** Baseline characteristics, PCa detection, and mortality within 20 yr by group and randomisation method (pre- and post-consent centres)

	Pre-consent centres^a^			Post-consent centres^b^		
	Never-attenders	Ever-attenders	Control arm	Never-attenders	Ever-attenders	Control arm
*n* (%)	10 966 (10)	33 802 (32)	61 223 (58)	1435 (2.6)	26 257 (47)	27 697 (50)
Age at randomisation (yr), median (IQR)	59 (55–63)	59 (55–63)	59 (55–63)	61 (58–65)	61 (58–65)	61 (58–65)
PCa detection at 20-yr, cumulative incidence (95% CI) [*n*]	8.3 (7.8–8.9) [914]	14 (14–15) [4762]	11 (11–11) [6819]	8.5 (7.0–9.9) [121]	15 (15–16) [3950]	9.9 (9.5–10) [2710]
PCa specific mortality at 20-yr, cumulative incidence (95% CI) [*n*]^c^	1.4 (1.2–1.7) [159]	0.97 (0.87–1.1) [328]	1.3 (1.2–1.4) [777]	1.1 (0.58–1.7) [16]	0.97 (0.85–1.1) [250]	1.2 (1.1–1.3) [328]

CI = confidence interval; IQR = interquartile range; PCa = prostate cancer.

a Sweden, Finland, Italy.

b Netherlands, Belgium, Spain, Switzerland.

c Rate ratios for never-attenders versus control arm and ever-attenders versus control arm per consent group are provided in Supplementary Table S2.

### 3.2 Screening attendance

**Fig. 1:**
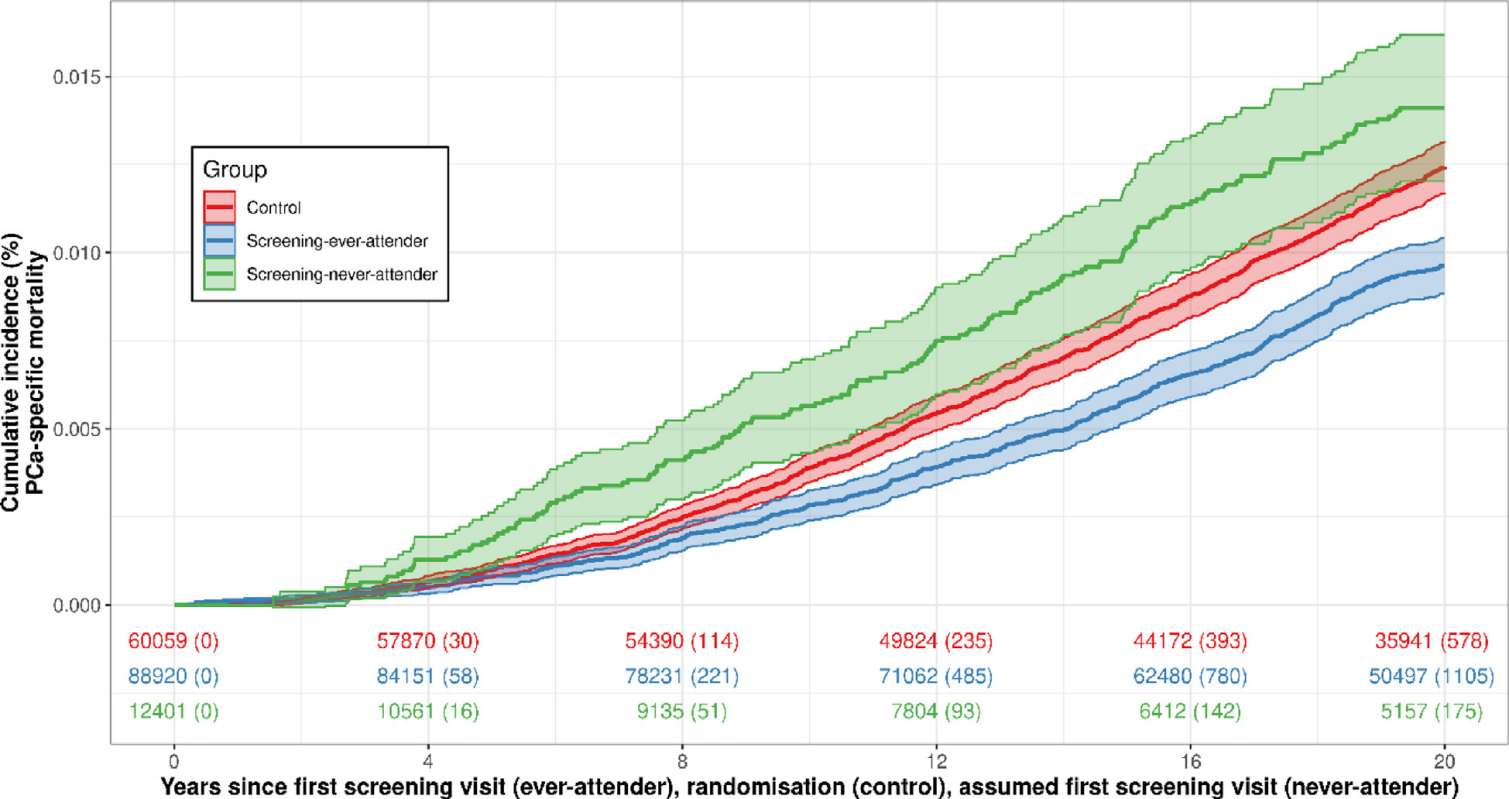
Cumulative incidence of prostate cancer (PCa) specific mortality per group. Including the total number at risk (cumulative number of PCa-specific mortality).

The cumulative PCSM at 20 yr of follow-up was 1.4% (95% CI, 1.2–1.6) for screening never-attenders, 1.2% (95% CI, 1.2–1.3) for the CA, and 1.0% (95% CI, 0.9–1.0) for screening ever-attenders (Fig. 1). The RR for PCSM in never-attenders compared to the CA was 1.39 (95% CI, 1.18–1.63; *p* < 0.001). In contrast, the RR for PCSM in ever-attenders compared to the CA was 0.77 (95% CI, 0.69–0.85; *p* < 0.001).

### 3.3 Exploratory analysis

Additionally, an off-note comparison of ever-attenders to never-attenders within the SA - reflecting groups most likely to attend or not attend screening in an actual screening programme - showed an RR of 0.55 (95% CI, 0.47–0.65; *p* < 0.001) for ever-attenders compared to never-attenders.

### 3.4 Sensitivity analyses

Table 2 shows the baseline characteristics and the cumulative incidence of PCa and PCSM at 20 yr by type of randomisation (pre vs post consent). An interaction between the three groups and the type of randomisation on PCSM did not statistically improve the fit of the model compared to main effects alone (*p* = 0.4). In addition, including the type of randomisation next to the ‘original’ Poisson regression did not statistically improve model fit (*p* = 0.5). Subgroup-specific RRs per consent group are presented in Supplementary Table S2 and should be interpreted with caution, given the limited statistical power within each stratum and the nonsignificant interaction test. Additionally, adjusting for centre as a covariate in the primary Poisson regression model did not substantially affect the main estimates (Supplementary Table S1), nor did the application of a symmetric immortal time bias correction to all groups (Supplementary Table S3).

## 4 Discussion

In this analysis of screening never-attenders in a large population-based European PCa randomised screening trial (ERSPC) with long-term follow-up, we observed that men in the intervention arm who were offered screening but did not attend screening showed an almost 40% higher mortality from PCa compared to those randomised to the CA who were not offered screening. Secondly, we observed a 23% lower PCa mortality for men who actually attend screening (SA ever-attenders) as compared to men in the CA.

### 4.1 Screening never-attenders

Never-attenders in the ERSPC were invited to participate in screening but opted not to. While both screening never-attenders and men in the CA did not undergo organised screening, we observed higher PCSM for the former subgroup than the latter. However, as additional data on potential confounders for the observed association are lacking, causality cannot be established.

Nevertheless, based on what is known from the literature, a plausible explanation for the observed association could be that screening never-attenders may disproportionately represent a subgroup with a relatively higher percentage of men with lower socioeconomic status (SES) and unfavourable health-related behaviours or access. Although individual-level SES data for all men randomised in the ERSPC are lacking, some information is known from previous publications. An analysis of the Finnish ERSPC centre showed that men with higher SES were more likely to participate than men with a lower SES, according to all SES indicators (educational level, income, and home ownership) [19]. Screening attendance in the Swedish ERSPC ranged from 45% to 83% across sociodemographic subgroups. The highest proportion of men attending was seen in the medium/high education (80%) and cohabiting (83%) subgroups [20]. A study of 500 men refusing an invitation to the ERSPC Rotterdam centre between 1995–1996 showed that, compared with attenders, refusers were older, less often married, had a lower level of education, less knowledge about PCa, and a less positive attitude towards screening [21]. Attenders of screening are known to be generally healthier and more health-conscious than nonattenders (healthy screenee bias), which was also reflected in higher overall mortality for nonattenders in previous analyses of the Swedish ERSPC [22]. Another study of the Finnish ERSPC showed that nonparticipants, as compared to participants, were more commonly current smokers. Nonparticipation was mainly due to practical reasons, such as forgetting the appointment, rather than negative attitudes toward screening [23].

These ERSPC-specific findings are consistent with a wider body of literature. Established cancer screening programmes for colorectal, breast, and cervical cancer demonstrate socioeconomic inequalities in participation [24], [25], [26], [27]. Similar patterns are observed in unorganised PCa early detection settings, where a higher SES is typically linked with a greater incidence of low- and intermediate-risk PCa (likely reflecting increased diagnostic activity) and a lower incidence of advanced PCa and PCSM [19], [28]. Conversely, men with lower SES are more likely to present with advanced disease and experience higher mortality [19], [28]. Secondary analyses of the Prostate, Lung, Colorectal, and Ovarian (PLCO) Cancer Screening trial demonstrated that a nonadherence behaviour profile was associated with higher overall mortality (excluding deaths from cancers studied in the trial) [29]. These findings underscore how social determinants of health shape participation in screening trials and similarly influence uptake in real-world settings [30].

Some studies have suggested that organising screening may help reduce socioeconomic inequalities by proactively inviting participants with less health care usage [20], [31], [32]. However, others suggest that organising screening using traditional methods of invitation (eg, an invitation letter) alone is insufficient as attendance is generally higher in higher SES strata [19], [33], [34], [35]. In Sweden, for example, the transition from an unorganised to an organised population-based PCa testing (OPT) programme in 2020 improved but did not eliminate disparities. Recent analyses showed that socioeconomic factors such as low income or living alone were associated with lower participation in PSA testing in both the organised OPT and the unorganised pre-OPT setting [33].

Altogether, these findings indicate that simply inviting men to participate in screening is not enough. This is not only a problem for current cancer screening programmes across Europe, as seen in the declining trend in overall participation rates, but more importantly because of the inequities within the group that chooses to attend- or not.

Off-trial PSA testing could have occurred in both screening never-attenders and men in the CA, a phenomenon known as contamination or opportunistic testing. The exact level of contamination in the ERSPC is unknown. However, the frequency of PSA testing in the initial years of the ERSPC trial (most relevant to the endpoint) has previously been estimated to range from 7% to 37%, depending on the definition, estimation method, and centre [36], [37], [38], [39]. It is plausible that contamination was higher in the CA than in the screening never-attender group, as men who actively declined the offered screening invitation are less likely to seek PSA testing outside the trial*.* Contamination is expected to be lower in centres using pre-consent randomisation, as men in the CA are never invited to the screening study and may be less aware of PSA testing opportunities. Overall, these levels of CA contamination may have contributed to the observed association - although they are unlikely to fully explain the observed difference in PCSM (RR, 1.39).

### 4.2 Screening attenders

In addition to the screening never-attenders, we estimated PCa mortality in men who actually attended screening. As expected, the observed PCSM reduction in this group is larger than the effectiveness reported in the intention-to-screen (ITS) analysis.

For screening ever-attenders compared to the CA, we observed an RR of 0.77 (95% CI, 0.69–0.85; *p* < 0.001). Prior ITS analyses - including all men in the SA regardless of attendance - reported relative reductions in PCSM of 21% at 13 yr [15] and 20% at 16 yr follow-up [16]. It is important to acknowledge that the method described by Cuzick et al. [40] was not used, as this was not the primary objective of this paper. Nevertheless, this descriptive observation is directionally consistent with prior analyses that estimated efficacy after correction for noncompliance and contamination within the ERSPC [41], [42], [43], [44].

Furthermore, comparison of screening ever-attenders and screening never-attenders within the SA showed a 45% lower PCSM for attenders. However, this cannot be interpreted as screening effects as it reflects the composite effect of both selective participation and the intervention itself.

### 4.3 Implications and recommendations

While participation in screening, in principle, remains a personal choice, it is critical that all individuals are equally informed about the option to participate and have access to the necessary information to make an informed decision. Current and future initiatives need to better identify and understand the differences between ever- and never-attenders. This knowledge could inform the further development, refinement, and implementation of interventions aimed at addressing underlying structural and behavioural barriers. Valuable lessons can be drawn from other established programmes, such as the use of personalised invitations [45] or interventions targeting multiple participation barriers [46], [47], both of which have shown promise in increasing participation. Ma et al [14] recently emphasised the importance of targeted interventions for first-round nonparticipants in a population-based breast cancer screening programme, as these women are less likely to participate in subsequent rounds and are at increased risk of breast cancer-specific mortality. However, these studies primarily focus on women. Therefore, in the context of PCa specifically, attention should be directed towards nonattendance and the development of targeted interventions within prospective screening initiatives. Nevertheless, even with improved understanding, the implementation of stratified approaches at a population scale will remain one of the most pressing challenges ahead.

### 4.4 Limitations

A limitation of this analysis is the lack of individual-level data on socioeconomic factors and other potential determinants of (non) attendance within the ERSPC, for which their contribution to the observed associations could not be evaluated. As such, we cannot make any causal statements. The use of different index times across groups to address immortal time bias represents a design choice that introduces some asymmetry. Sensitivity analysis confirmed minimal impact on the results (Supplementary Table S3). Furthermore, the exact level of off-trial PSA testing in both the never-attender and control groups is unknown, and the contribution of differential contamination to the observed association therefore remains uncertain.

## 5 Conclusion

Three key messages can be drawn from the results. The ‘good’ message is that the observed PCSM reduction for men who actually attend screening is larger than that reported in the ITS analysis. The ‘bad’ message is that for nonattenders of screening, we observed higher PCSM than in the CA. The ‘ugly’ message is that this group with the worst observed outcome is notably the group that was offered screening but opted not to attend. This underscores the need for future initiatives to gain a deeper understanding of screening (non) attendance and its effect on organised programmes, to ultimately strive for equitable screening benefits and minimise further contribution to health disparities.

  ***Author contributions:*** Renée C.A. Leenen had full access to all the data in the study and takes responsibility for the integrity of the data and the accuracy of the data analysis.

  *Study concept and design:* Leenen, Mulder, Remmers, de Vos, Talala, Hugosson, Arnsrud-Godtman, Den Hond, Villers, Pionas, Llanes, Lujan, Gorini, Puliti, Kwiatkowski, Wyler, Bangma, Schroder, Auvinen, Roobol.

*Acquisition of data:* Talala, Hugosson, Arnsrud-Godtman, Den Hond, Villers, Pionas, Llanes, Lujan, Gorini, Puliti, Kwiatkowski, Wyler, Bangma, Schroder, Auvinen, Roobol.

*Analysis and interpretation of data:* Leenen, Mulder, Remmers, de Vos.

*Drafting of the manuscript:* Leenen.

*Critical revision of the manuscript for important intellectual content:* Mulder, Remmers, de Vos, Talala, Hugosson, Arnsrud-Godtman, Den Hond, Villers, Pionas, Llanes, Lujan, Gorini, Puliti, Kwiatkowski, Wyler, Bangma, Schroder, Auvinen, Roobol.

*Statistical analysis:* Leenen, Mulder, Remmers.

*Obtaining funding:* Talala, Hugosson, Arnsrud-Godtman, Den Hond, Villers, Pionas, Llanes, Lujan, Gorini, Puliti, Kwiatkowski, Wyler, Bangma, Schroder, Auvinen, Roobol.

*Administrative, technical, or material support:* Remmers.

*Supervision*: Mulder, Remmers, de Vos, Roobol.

*Acknowledgements:* Acknowledged persons are presented in Supplementary 1 (ERSPC investigators).

  ***Financial disclosures:*** Renée C.A. Leenen certifies that all conflicts of interest, including specific financial interests and relationships and affiliations relevant to the subject matter or materials discussed in the manuscript (eg, employment/affiliation, grants or funding, consultancies, honoraria, stock ownership or options, expert testimony, royalties, or patents filed, received, or pending), are presented in Supplementary 2 (ERSPC grants per centre).

  ***Funding/Support and role of the sponsor:*** ERSPC was supported by grants (Supplementary 2 presents ERSPC grants per centre). The sponsors had no role in the design and conduct of the study; collection, management, analysis, and interpretation of the data; and preparation, review, and approval of the manuscript.

## Appendix A Supplementary data

Supplementary 1 ERSPC investigators.

Supplementary 2 ERSPC Grants per centre.
