# Supplementary material for: Prostate Cancer Mortality in Men not Attending a Population-based Screening Program: The ‘Good’, the ‘Bad’, and the ‘Ugly’ After 20-yr Follow-up in the European Randomised study of Screening for Prostate Cancer
Source: Eur Urol Open Sci. 2026 Jul 30;91:59–66. doi: 10.1016/j.euros.2026.06.009 (PMC13452280; doi:10.1016/j.euros.2026.06.009)
Supplement: Supplementary file 1 [file mmc1.docx]

**Appendix : ERSPC investigators**

The following people are being acknowledged for their contribution to the ERSPC study.

**The Netherlands:**

Dr. W.J. Kirkels, urologist

Dr. J.B.W Rietbergen, urologist

Dr. I.W. van der Cruijsen, urologist

Dr. R. Raaijmakers, urologist

Dr. S.H. de Vries, urologist

Dr. S. Roemeling, urologist

Dr. C. Gosselaar, general practicioner

Dr. T. Wolters, urologist

Dr. R.C.N. van den Bergh, urologist

Dr. P.J. van Leeuwen, urologist

Dr. M. Bul, PhD urologist

Dr. X. Zhu, PhD urologist

Dr. L.P. Bokhorst, radiologist

Dr. A.R.Alberts, urologist

Dr. F-J. Drost, GP

Dr. J.F.M. Verbeek, Radiologist

Dr. D.F. Osses, Urologist

Dr. H.B. Luiting, Urologist

Dr. R. Hogenhout, Urologist

Dr. I. I. de Vos, Urologist

Drs. R.C.A. Leenen, PhD student

Drs. J. J. Lodder, PhD student

Drs. M.J. van Harten, PhD student

Drs. S.F. Westerhout, PhD student

Drs. F.B. Denijs, PhD student

E.F.H. Mulder, MSc student

Dr. L.D.F. Venderbos, quality of life researcher

Dr. K. Beyer, postdoctoral researcher

Dr. H.A. van Vugt

Drs. G. Yurdakul, urologist

Drs. A. Boeken-Kruger, urologist

Drs. C. Wijburg, urologist

Drs. M. Forouzanfor, urologist

Drs. M. de Boer, urologist

Dr. R. Postma, pathologist

Dr. A.N. Vis, urologist

Prof. Dr. Th van der Kwast, pathologist

Dr. R. Hoedemaeker, pathologist

Prof. Dr. G.J.L.H. van Leenders, pathologist

Dr. R. van Schaik, clinical chemistry

Prof. Dr. P.J. van der Maas, epidemiologist

Prof. Dr. H.J. De Koning, epidemiologist

Dr. E.A. Heijnsdijk, epidemiologist

Dr. S. Otto, epidemiologist

Dr. G. Draisma, statistician

Dr. P. Beemsterboer, epidemiologist

Dr. I. Korfage, epidemiologist

Dr. R. Boer, informatician/mathematician

Dr. M. Wildhagen, statistician

Drs. D. Nieboer, statistician

Drs. W. Merkelbach, COD committee

Dr. W. Hoekstra, COD committee

Dr. J. Blom, COD committee

Dr. T. Lock, COD committee

Dr. A. Noordzij, COC committee

Drs. RAM Damhuis, epidemiologist, cancer registry

Drs. A. Reedijk, epidemiologist, cancer registry

Dr. R. Kranse, statistician, cancer registry

Mrs. D.W. Roobol, data management

Mr. W. Roobol, website management

Mrs. E. van den Berg, administrative support

Mr. G-J de Zwart, echografist

Mrs. C.G.A.M. Franken-Raab, data management

Mrs. M. van Slooten-Midderig, data management

Mrs. A. Smit, medical assistant

Mrs. V van der Drift, data management

Mrs. E. de Bilde, data management

Mrs. M den Rooijen, data management

Mrs. L. Mani, medical assistant

Mrs. M. Visser-van Dongen, data management

Mrs. H. Versteeg-Leenheer, data management

Mrs. B. Zoutendijk, data management

Mrs. H. van Meurs, echografist

Mrs. A E. de Bruijn, secretary

**Belgium:**

Prof. Dr. L.Denis, professor of Urology

H. Devriendt Dr.Sc, director, Provincial Institute Hygiene

Dr. B. Standaert, director, Provincial Institute Hygiene

G. Thijs, Data Manager, Provincial Institute Hygiene

A. Hermans, Data Manager, Provincial Institute Hygiene

K. D'Hooge, Data Manager, Provincial Institute Hygiene

Dr. H. Neels, lab Biochemistry, ZNA Antwerp City Hospitals

Dr. A. Wauters, lab Biochemistry, ZNA Antwerp City Hospitals

Dr. I. Neetens, lab Pathology, ZNA Antwerp City Hospitals

Dr. M. Kockx, lab Pathology, ZNA Antwerp City Hospitals

Prof. F. Keuppens, University Hospital Brussels Urology

Dr. J. Braeckman, University Hospital Brussels Urology

Dr. F. Govaerts, General Practice Associations, WVVH, UHAK, DOMUS, HAVAC

Dr. B. Spinnewijn, General Practice Associations, WVVH, UHAK, DOMUS, HAVAC

Dr. M. Van Hoey, General Practice Associations, WVVH, UHAK, DOMUS, HAVAC

Dr. T. Putzeys, General Practice Associations, WVVH, UHAK, DOMUS, HAVAC

M. De Coninck, chair, Oncologic Centre Antwerp

B. Dourcy-Belle-Rose, Coordinator, Oncologic Centre Antwerp

A. Vancauwenbergh, Secretary, Oncologic Centre Antwerp

Prof. Dr. J.W. Coebergh, COD committee

Dr. H. Verhaegen, COD committee

Dr. P. Van Vliet, COD committee

Mr. L. Van Herck, Administrative director, City of Antwerp

**Sweden:**

Dr. Gunnar Aus, Urologist, former voting member

Maria Nyberg, study nurse

Svante Bergdahl, Urologist

Pär Lodding, Urologist

Ali Khatami, Urologist

Johan Stranne, Urologist

Helén Ahlgren, data manager, study secretary

Gun-Britt Eriksson, Laboratory technician

Charlotte Becker, MD, PhD, Clinical chemistry

Sigrid Carlsson, Dr. PhD

Anna Grenabo, Urologist

Dr. Maria Franlund, Urologist

Silas Pettersson, COD committee

Eberhard Varenhorst. COD committee

Bo-Johan Norlén, COD committee

Björn Zackrisson, Urologist

Erik Holmberg, Statistician and database manager

Roland Frösing, Urologist

Erik Pileblad, Urologist

Carl-Gustaf Pihl, Pathologist, pathology committee

Dr. Vasiliki Spyratou, Pathologist, pathology committee

J.Adolfsson, Urologist

H.Lilja, clinical chemist

U-H Stenman, clinical chemist

**Finland:**

Paula Kujala, MD, Professor, pathologist, Fimlab Laboratories

Kimmo Taari, MD, Professor, urologist, University of Helsinki and Helsinki University Hospital

Jani Raitanen, statistician, Tampere University

Tuomas Kilpeläinen, MD, PhD, urologist, University of Tampere and Tampere University Hospital

Neill Booth, PhD, health economist, Tampere University

Tomi Pakarainen, MD, PhD, urologist, Tampere University Hospital

Juho Pylväläinen, MD, PhD student, Helsinki University Hospital

Johanna Schleutker, Professor of medical genetics, University of Turku

Teemu Murtola MD, Professor, urologist, University of Tampere and Tampere University Hospital

Aino Siltari, PhD, epidemiologist, Tampere University

Matti Hakama, Professor, Finnish Cancer Registry

Patrik Finne, MD, Professor, Helsinki University Hospital

Hanna Vasarainen, MD, PhD, urologist, Tampere University Hospital

Thea Veitonmäki, MD, PhD, urologist, Tampere University Hospital

Kalle Kaapu, MD, PhD, Tampere	University Hospital

Pekka Rissanen, Professor, Tampere University

Marita Laurila, pathologist, pathology committee, Tampere University Hospital

Mirja Ruutu, MD, Professor, urologist, Helsinki University Hospital

Jussi Aro, MD, PhD, urologist, COD committee, Helsinki University Hospital

Harri Juusela, MD, PhD, urologist, Helsinki University Hospital, Jorvi Hospital

Markus Mildh, MD, PhD, urologist, Helsinki University Hospital, Peijas-Rekola Hospital

Jorma Lahtela, MD, Professor, COD committee, Tampere University Hospital

Pekka J Karhunen, MD, Professor, COD committee, University of Tampere

Ulf-Håkan Stenman, MD, Professor, Helsinki University Hospital

Tuukka Mäkinen, MD, urologist, Tampere University Hospital

Antti Rannikko, MD, Professor, urologist, Helsinki University Hospital

Sakari Rannikko, MD, Professor, urologist, Helsinki University Hospital

Nea Malila, Professor, Finnish Cancer Registry

Liisa Määttänen, PhD, database management, Finnish Cancer Registry

Sanna Kuivalainen, research nurse, Finnish Cancer Registry

Tiina Karhunen, research nurse, Finnish Cancer Registry

Irja Erkkilä, research nurse, Finnish Cancer Registry

**Italy:**

Mario Cappellini, COD Committee

Simonetta Bianchi, COD Committee

Claudio Lombardi, COD Committee

Alessandro Bussotti, COD Committee

Paolo Bastiani, COD Committee

Co-operators:

Rita Bonardi

Antonia Mazzotta

Dusca Bartoli

Liana Bonfrisco

Emanuele Crocetti

Francesca Martinelli

Paola Zendron

Paola Frullini

Simonetta Di Lollo, Pathology committee

Tiziana Rubeca

**Spain:**

Antonio Berenguer. Servicio de Urologia. Hospital de Madrid Norte Sanchinarro. Madrid, Spain.

Jose A. Rodriguez. Instituto Nacional de Estadistica. Madrid, Spain.

Benita Aybar. Instituto Nacional de Estadistica. Madrid, Spain.

Luis Llanes. Hospital Universitario de Getafe. Madrid, Spain.

Alba María García-Cano. Hospital Universitario de Getafe. Madrid, Spain.

**Switzerland:**

Kantonsspital Aarau, Aarau

Prof. Dr. Reto Tscholl, Urologist, local COD Committee

PD Dr. med. Marco Randazzo, Urologist

Dr. med. Daniel Seiler, Urologist

Dr. med. Martin Baumgartner, Urologist

Prof. Dr. med. Andreas Huber, Centre of Laboratory Medicine

Dipl. Pharm. Roberto Herklotz, Centre of Laboratory Medicine

Prof. Dr. Bernhard Stamm, Pathologist

Dr. med. Martin Wernli, Oncologist

Prof. Dr. Stephan Bodis, Radiooncologist

Prof. Dr. Rainer Grobholz, Pathologist

Mrs. Elsbeth Woodtli, study nurse/ data management

Mrs. Lydia Schärer, study nurse/ data management

Mrs. Cornelia Kaspar, study nurse/ data management

Mr. Heinz Mathis, logistics

University Hospital Basel, Basel, Institute for Pathology, Division of Cytology

Prof. Dr. Lukas Bubendorf, Review Pathologist, Pathology Committee

Kantonsspital Baden, Baden

Dr. med. Kurt Lehmann, Urologist

PD Dr. med. Lukas Hefermehl, Urologist

Dr. Hans-Ruedi Schmid, Central Laboratory

Aargau Association of Medical Doctors, Baden

Dr. med. Hans-Ulrich Iselin, General Practitioner, cooperation with GPs in Aargau

University Hospital Zürich, Zürich

Prof. Dr. Johann Steurer, Internist, local COD Committee

Prof. Dr. Burkhardt Seifert, Division of Biostatistics

Privatklinik Bethanien, Zürich

PD Dr. Michael Kurrer, Pathologist, local COD Committee

Department of Urology, Academic Hospital Braunschweig, Braunschweig, Germany

Prof. Dr. Peter Hammerer, Urologist, local COD Committee

**France:**

Bernard Malavaud, urologist

François Iborra, urologist

Xavier Rebillard, urologist

Sandra Malavaud, epidemiologist

Pascale Grosclaude, epidemiologist

Brigitte Tretarre, epidemiologist

Vincent Bataille, data manager

Faiza Bessaoud, dara manager

**The United Kingdom:**

Datamanagers of the ERSPC central database:

J.Hemming

P.Coulson

D.Coleman

Prof. Dr. Ph.Smith, urologist

Dr. S.Moss, epidemiologist
