# Supplementary material for: Prostate Cancer Mortality in Men not Attending a Population-based Screening Program: The ‘Good’, the ‘Bad’, and the ‘Ugly’ After 20-yr Follow-up in the European Randomised study of Screening for Prostate Cancer
Source: Eur Urol Open Sci. 2026 Jul 30;91:59–66. doi: 10.1016/j.euros.2026.06.009 (PMC13452280; doi:10.1016/j.euros.2026.06.009)
Supplement: Supplementary file 2 [file mmc2.docx]

**Grants received per centre**

*The Netherlands*:

The Dutch Cancer Society (KWF 94-869, 98-1657, 2002-277, 2006-3518, 2010-4800); The Netherlands Organisation for Health Research and Development (ZonMW-002822820, 22000106, 50-50110-98-311, 62300035), unrestricted grants from the SWOP (Prostate Cancer Research Foundation) and the Abe Bonnema Stichting.

*Belgium*:

Europe against Cancer, Flemish Ministry of Welfare, Public Health and Family, Province and City of Antwerp, Public Centre for Social Welfare Antwerp

*Sweden*:

Abbott Pharmaceuticals, Sweden, Af Jochnick’s foundation, Catarina and Sven Hagstroms family foundation, Gunvor and Ivan Svensson’s foundation, Johanniterorden, King Gustav V Jubilée Clinic Cancer Research Foundation, Sahlgrenska University Hospital, Schering Plough, Sweden, Swedish Cancer Society, Swedish Research Counsil, Wallac Oy, Turkku, Finland.

Personal grants

Hans Lilja and Sigrid Carlsson were supported in part by a Cancer Center Support Grant to Memorial Sloan Kettering Cancer Center [P30 CA008748] from the National Institutes of Health/National Cancer Institute (NIH/NCI) and the Sidney Kimmel Center for Prostate and Urologic Cancers, David H. Koch through the Prostate Cancer Foundation. H.L was also supported in part by the National Institute for Health Research (NIHR) Oxford Biomedical Research Centre Program in the UK, the Swedish Cancer Society (CAN 2017/559), and the Swedish Research Council (VR-MH project no. 2016-02974).

*Finland*:

Academy of Finland (Grant no. 123054, 261205, 260931), Competitive Research Funding of the Tampere University Hospital (Grant no. 9K131, 9L103, 9M102, 9N078, and 9P053), Nordic Cancer Union, The Cancer Society of Finland

*Italy*:

Italian League for the Fight aganist Cancer - LILT Lega Italiana per la Lotta contro i Tumori, Italian Association for Cancer Research - AIRC Associazione Italiana Ricerca sul Cancro, National Reasrch Council - CNR Consiglio Nazionale delle Ricerche, Tuscany Region - Regione Toscana

*Spain:*

  The Spanish "Fondo de Investigación Sanitaria": 96/0248, 99/0245, 02/0732, 06/0831.

·         The Spanish ‘Fundación para la Investigación en Urología de la Asociación Española de Urología’, grant Pedro Cifuentes 2017.

·         The Spanish ‘Asociación Madrileña de Urología’, grant Plan de Investigación 2017.

*Switzerland*:

The Horten Foundation, Aargau Cancer League, Swiss Cancer League (Grant Nr KFS 787-2-1999 and 01112-02-2001), Health Department of Canton Aargau, Prostate Cancer Research Foundation, Baugarten Foundation and the Messerli Foundation.

*France:*

Institut National du Cancer convention 12-2008 Convention : 07/3D1616/SPC-111-13/NG-NC Appel à projets 2007 : Santé publique et Cancers. Ligue Nationale Contre le cancer PROJET N° PRE08/AV – CONV. DRC 2009/119. Ligue Nationale Contre le cancer PROJET N°: EPDCD14646– DRC n° 2014/620- N° PRE2014.LNCC/AV

*International coordination*:

European Union Grants SOC 95 35109, SOC 96 201869 05F022, SOC 97 201329, SOC 98 32241, the 6th Framework Program of the EU: PMark:LSHC-CT-2004-503011;

European Association of Urology supports the maintenance of the Central Database

*Unconditional grants*:

Beckman-Coulter-Hybritech Inc
