# Supplementary material for: Prostate Cancer Mortality in Men not Attending a Population-based Screening Program: The ‘Good’, the ‘Bad’, and the ‘Ugly’ After 20-yr Follow-up in the European Randomised study of Screening for Prostate Cancer
Source: Eur Urol Open Sci. 2026 Jul 30;91:59–66. doi: 10.1016/j.euros.2026.06.009 (PMC13452280; doi:10.1016/j.euros.2026.06.009)
Supplement: Supplementary file 3 [file mmc3.docx]

**Supplementary table S1** **Rate ratios for PCSM adjusted for centre**

|  | RR (95%CI) | p-value |
| --- | --- | --- |
| Never-attender vs control | 1.36 (1.15-1.59) | <0.001 |
| Attenders vs control | 0.77 (0.69-0.86) | <0.001 |
| Attender vs never-attender | 0.57 (0.48-0.68) | <0.001 |

**Supplementary Table S2 Rate ratios for PCSM per consent group**

|  | Pre-consent centres* |  | Post-consent centres** |  |
| --- | --- | --- | --- | --- |
|  | **RR (95%CI)** | **p-value** | **RR (95%CI)** | **p-value** |
| Never-attender vs control | 1.41 (1.18-1.68) | <0.001 | 1.08 (0.63-1.72) | 0.8 |
| Attenders vs control | 0.75 (0.65-0.86) | <0.001 | 0.80 (0.68-0.94) | 0.006 |
| ** Sweden, Finland, Italy, **Netherlands, Belgium, Spain, Switzerland* | | | | |

**Supplementary Table S3 Sensitivity analysis immortal time bias correction - symmetric immortal time correction applied to all groups**

|  | Cumulative PCSM at 20-yr % (95%CI) | RR vs control (95%CI) |
| --- | --- | --- |
| Never-attender | 1.41 (1.20-1.62) | 1.38 (1.17-1.62) |
| Attender | 0.97 (0.89-1.05) | 0.76 (0.68-0.84) |
| Control | 1.25 (1.17-1.32) | Reference |
